# A methodology to assess the intrinsic discriminative ability of a distance function and its interplay with clustering algorithms for microarray data analysis

**DOI:** 10.1186/1471-2105-14-S1-S6

**Published:** 2013-01-14

**Authors:** Raffaele Giancarlo, Giosué Lo Bosco, Luca Pinello, Filippo Utro

**Affiliations:** 1 Dipartimento di Matematica ed Informatica, Universitá di Palermo, Via Archirafi 34, 90123 Palermo, Italy; 2 Department of Biostatistics at Dana-Farber Cancer Institute and Harvard School of Public Health, 44 Binney Street, Boston, Massachusetts 02115, USA; 3 Computational Genomics Group, IBM T.J. Watson Research Center, 1101 Kitchawan Road, Route 134, Yorktown Heights, N.Y. 10598, USA

## Abstract

**Background:**

Clustering is one of the most well known activities in scientific investigation and the object of research in many disciplines, ranging from statistics to computer science. Following Handl *et al*., it can be summarized as a three step process: (1) choice of a distance function; (2) choice of a clustering algorithm; (3) choice of a validation method. Although such a purist approach to clustering is hardly seen in many areas of science, genomic data require that level of attention, if inferences made from cluster analysis have to be of some relevance to biomedical research.

**Results:**

A procedure is proposed for the assessment of the discriminative ability of a distance function. That is, the evaluation of the ability of a distance function to capture structure in a dataset. It is based on the introduction of a new external validation index, referred to as *Balanced Misclassification Index *(*BMI*, for short) and of a nontrivial modification of the well known Receiver Operating Curve (ROC, for short), which we refer to as Corrected ROC (CROC, for short). The main results are: (a) a quantitative and qualitative method to describe the intrinsic separation ability of a distance; (b) a quantitative method to assess the performance of a clustering algorithm in conjunction with the intrinsic separation ability of a distance function. The proposed procedure is more informative than the ones available in the literature due to the adopted tools. Indeed, the first one allows to map distances and clustering solutions as graphical objects on a plane, and gives information about the bias of the clustering algorithm with respect to a distance. The second tool is a new external validity index which shows similar performances with respect to the state of the art, but with more flexibility, allowing for a broader spectrum of applications. In fact, it allows not only to quantify the merit of each clustering solution but also to quantify the agglomerative or divisive errors due to the algorithm.

**Conclusions:**

The new methodology has been used to experimentally study three popular distance functions, namely, Euclidean distance *d*_2_, Pearson correlation *d_r _*and mutual information *d_MI_*. Based on the results of the experiments, we have that the Euclidean and Pearson correlation distances have a good intrinsic discrimination ability. Conversely, the mutual information distance does not seem to offer the same flexibility and versatility as the other two distances. Apparently, that is due to well known problems in its estimation. since it requires that a dataset must have a substantial number of features to be reliable. Nevertheless, taking into account such a fact, together with results presented in Priness *et al*., one receives an indication that *d_MI _*may be superior to the other distances considered in this study only in conjunction with clustering algorithms specifically designed for its use. In addition, it results that K-means, Average Link, and Complete link clustering algorithms are in most cases able to improve the discriminative ability of the distances considered in this study with respect to clustering. The methodology has a range of applicability that goes well beyond microarray data since it is independent of the nature of the input data. The only requirement is that the input data must have the same format of a "feature matrix". In particular it can be used to cluster ChIP-seq data.

## Background

Recently, medical and biological research has been deeply influenced by the advent of high throughput technologies such as microarrays and RNA-seq platforms. They enable the acquisition of data that are fundamental for research in several areas of the biological sciences such as understanding biological systems and diagnosis (e.g. [1]). A fundamental aspect of microarray data analysis consists of clustering gene expression data [2,3]. However, its application to post-genomic data has revealed to be rather *ad hoc*. That is the reason why there is hardly any consensus on the best distance function and clustering algorithm to be used for the different types of post-genomic data. As a consequence, the common practice is to use several different clustering techniques to analyse a dataset, and to resort to visual inspection and prior biological knowledge to select what is considered the most "appropriate" result. Clearly, this data analysis is highly subjective, implying obvious risks. Those observations have motivated Handl *et al*. [4] to write a seminal paper with the intent to show to both bioinformatics researchers and end-users some of the fundamental aspects of the clustering methodology. The main message of that paper is that clustering can be considered as a three step process: (1) choice of a distance function; (2) choice of a clustering algorithm and (3) choice of a methodology to estimate the statistical significance of clustering solutions. Points (2) and (3) lead into two well established and rich research areas in data analysis ranging from statistics to Computer Science. Although computational methods for the analysis of microarray data have witnessed an exponential growth, a few contributions have been given in trying to assess their merits [5]. As a result, the need for a comprehensive evaluation of the whole analysis process for microarray data is being recognized and a few benchmarking studies have appeared [6-8]. Unfortunately, point (1) has been hardly investigated regarding this new type of data and very few results on this topic are available (see [2,9,10] and references therein).

In this paper, we address point (1) by introducing a new qualitative and quantitative method to describe and assess the discriminative ability of a distance function alone and in conjunction with a clustering algorithm. Moreover, the methodology is also able to give indications about the bias of clustering algorithms with respect to distances. It is worth recalling that very little is known about this latter point, one of the difficulties being a fair comparison between the performance of a distance function and a clustering algorithm measured in terms of their classification ability. This point is discussed in detail in the *Methods *section. The overall methodology that is introduced here makes use of the ROC plane and the ROC curve [11] in order to define the new external clustering validation index *BMI *and the new CROC curve. The net effect is the delivery of a methodology that rigorously uses external knowledge in order to assess the performance of a distance function while granting a fair comparison with clustering solutions generated by a clustering algorithm. It is worth mentioning that previous approaches to this problem presented the shortcomings of being based only on internal indices [10], i.e., homogeneity and separation: Indeed, it is well known that external validation is more accurate than the internal one [12]. The remainder of this paper is organized as follows. The experimental set-up we have used and the results are presented in the next section. Then, some conclusions and directions of future research are offered next. Finally, the *Methods *section describes in detail the new methodology to assess the intrinsic separation ability of three distance functions, and its use in conjunction with clustering algorithms.

## Results and discussion

### Experimental setup

#### Datasets

Technically speaking, a *gold solution *GS for a dataset is a partition of the data in a number of classes known *a priori*. Membership of a class is established by assigning the appropriate class label to each element. This means that the partition of the dataset in classes is based on some external knowledge that leaves no ambiguity on the actual number of classes and on their composition in terms of class memberships. Moreover, is also important to state that there exist two main kinds of gold solution datasets, i.e., (i) the ones for which *an priori *division in to classes of the dataset is known; (ii) and the ones for which high quality partitions have been inferred by analyzing the data. Dudoit and Fridlyand [13] elegantly make clear that difference in a related study and we closely follow their approach here.

Each dataset is a matrix, in which each row corresponds to an element to be clustered and each column to an experimental condition. The nine datasets, together with the acronyms used in this paper, are reported next. For conciseness, we mention only some relevant facts about them. The interested reader can find additional information in Dudoit and Fridlyand [13] for the Lymphoma and NCI60 datasets, Di Gesú *et al*. [14] for the CNS Rat, Leukemia and Yeast datasets and in Monti *et al*. [15], for the remaining ones.

CNS Rat: It is a 112 × 17 data matrix, obtained from the expression levels of 112 genes during a rat's central nervous system development. The dataset was studied by Wen *et al*. [16] and they suggested a partition of the genes into six classes, four of which are composed of biologically, functionally-related genes. This partition is taken as the gold solution, which is the same one used for the validation of FOM [17].

Gaussian3: It is a 60 × 600 data matrix. It is generated by having 200 distinctive features out of the 600 assigned to each cluster. There is a partition into three classes and that is taken as the gold solution. The data simulates a pattern whereby a distinct set of 200 genes is up-regulated in one of the three clusters, and down-regulated in the remaining two.

Gaussian5: It is a 500 × 2 data matrix. It represents the union of observations from 5 bivariate Gaussians, 4 of which are centered at the corners of the square of side length λ, with the 5th Gaussian centered at (λ/2, λ/2). A total of 250 samples, 50 per class, were generated, where two values of λ are used, namely, λ = 2 and λ = 3, to investigate different levels of overlapping between clusters. There is a partition into five classes and that is taken as the gold solution.

Leukemia: It is a 38 × 100 data matrix, where each row corresponds to a patient with acute leukemia and each column to a gene. The original microarray experiment consists of a 72 × 6817 matrix, due to Golub *et al*. [18]. In order to obtain the current dataset, Handl *et al*. [4] extracted from it a 38 × 6817 matrix, corresponding to the learning set in the study of Golub *et al*. and, via preprocessing steps, they reduced it to the current dimension by excluding genes that exhibited no significant variation across samples. The interested reader can find details of the extraction process in Handl *et al*.. For this dataset, there is a partition into three classes and that is taken as the gold solution. It is also worthy of mention that Leukemia has become a benchmark standard in the cancer classification community [19].

Lymphoma: It is a 80 × 100 data matrix, where each row corresponds to a tissue sample and each column to a gene. The dataset comes from the study of Alizadeh *et al*. [20] on the three most common adult lymphoma tumors. There is a partition into three classes and it is taken as the gold solution. The dataset has been obtained from the original microarray experiments, consisting of an 80 × 4682 data matrix, following the same preprocessing steps detailed in Dudoit and Fridlyand [13].

NCI60: It is a 57 × 200 data matrix, where each row corresponds to a cell line and each column to a gene. This dataset originates from a microarray study in gene expression variation among the sixty cell lines of the National Cancer Institute anti-cancer drug screen [21], which consists of a 61 × 5244 data matrix. There is a partition of the dataset into eight classes, for a total of 57 cell lines, and it is taken as the gold solution. The dataset has been obtained from the original microarray experiments as described by Dudoit and Fridlyand [13].

Novartis: It is a 103 × 1000 data matrix, where each row corresponds to a tissue sample and each column to a gene. The dataset comes from the study of Su *et al*. [22] on four distinct cancer types. There is a partition into four classes and we take that as the gold solution.

Simulated6: It is a 60 × 600 data matrix. It consists of a 600-gene by 60-sample dataset. It can be partitioned into 6 classes with 8, 12, 10, 15, 5, and 10 samples respectively, each marked by 50 distinct genes uniquely up-regulated for that class. In addition, a list of 300 noise genes (i.e., genes having the same distribution within all clusters) are included. In particular, such genes are generated with decreasing differential expression and increasing variation, following the same distribution. Finally, the first block of 50 genes of the list is assigned to cluster 1, the second block to cluster 2 and so on. This partition into 6 classes is taken as the gold solution.

Yeast: It is a 698 × 72 data matrix, studied by Spellman *et al*. [23] whose analysis suggests a partition of the genes into five functionally-related classes, which is taken as the gold solution and which has been used by Shamir and Sharan for a case study on the performance of clustering algorithms [24].

#### Distances

Let  be a set. A function δ:X×X→ℝ is a *distance *(or *dissimilarity*) on  if, ∀ x, y ∈ , it satisfies the following three conditions:

1. *δ*(**x, y**) ≥ 0 (*non*-*negativity*);

2. *δ*(**x, y**) = *δ*(**y, x**) (*symmetry*);

3. *δ*(**x, x**) = 0;

In the case of microarray data,  = ${\mathbb{R}}^{m}$, i.e. each data point $\vec{x}$ is a vector in *m*-dimensional space. Note that a dataset **X **is a finite subset of , |**X**| = *n*. One can categorize distance functions according to three broad classes: *geometric, correlation*-*based *and *information*-*based*. Functions in the first class capture the concept of *physical *distance between two objects. They are strongly influenced by the magnitude of change in the measured components of vectors $\vec{x}$ and $\vec{y}$, making them sensitive to noise and outliers. Functions in the second class capture dependencies between the coordinates of two vectors. In particular, they usually have the benefit of capturing positive, negative and linear relationships between two vectors. Functions in the third class are defined via well known quantities in information theory such as entropy and mutual information [25]. They have the advantage of capturing statistical dependencies between two discrete data points, even if they are not linear. Unfortunately, when one tries to apply them to points in ${\mathbb{R}}^{m}$, a suitable discretization process must be carried out, known as *binning*, which usually poses some non-trivial challenges. For our experiments, we have considered the Euclidean distance, the Pearson correlation and Mutual Information since they are excellent representatives of the three categories described above. Indeed, they have been shown to be the most suitable for microarray data [9]. For the convenience of the reader, they are defined in the *Methods *section.

In what follows, we refer to distance and dissimilarity functions with the generic term distance functions.

### Algorithms and hardware

In our experiments, we have chosen K-means among *Partitional Methods*, and Average Link, Complete Link and Single Link among the *Hierarchical Methods *clustering algorithms. The details of those algorithms are not reported here and the interested reader can find a detailed description of them in [26]. Of course, each of the above mentioned algorithms has already been used for data analysis of microarray data, e.g. [14,27-29]. All experiments were performed on several state-of-the-art PCs.

### Evaluating the performance of distance functions via the BMI index and the CROC curve

In order to shed light on the proper choice of a distance function for clustering of microarray data, one needs to address the following points:

(A) Assessment of the intrinsic separation ability of a distance. That is, how well a distance discriminates independently of its use within a clustering algorithm.

(B) Assessment of the predictive clustering algorithm ability of a distance. That is, which distance function grants the best performance when used within a clustering algorithm.

(C) The interplay between (A) and (B).

Points (A) and (B) have been studied before (see [9] and references therein) with some useful insights. Unfortunately, very little is known about (C), one of the difficulties being a fair comparison between the performance of a distance function and a clustering algorithm measured in terms of classification ability (the technical details regarding this point are in the *Methods *section). We address this latter problem by introducing the *BMI *and the CROC curve. Technically, the idea is to map clustering solutions on the ROC plane and then to design a procedure that allows a fair comparison of distance functions and clustering algorithms via that mapping. Although the full details are given in the *Methods *section, it results convenient here to outline our approach. To this end, we need to introduce some terminology and recall some definitions. Given a clustering solution *C *= {*c*_1_, ...,*c_r_*}, it can be represented by a binary matrix *J*, referred to as connectivity matrix, where each entry of *J *is defined as follows:

(1) $$J\left(i,j\right)=\left\{\begin{array}{cc} 1 & \text{if }{\vec{x}}_{i}\text{ and }{\vec{x}}_{j}\text{ belong tothe same cluster,}\\ 0 & \text{otherwise}\text{.} \end{array}\right)$$

Note that an important property of the connectivity matrix is transitivity, i.e. ∀*i, j, k *such that *J*(*i, j*) = *J*(*j, k*) = 1, then *J*(*i, k*) = 1. This is straightforward since a clustering solution is a partition of the dataset, so that two different clusters have always empty intersection. A useful tool to assess the performance of a classifier, not necessarily binary, is the *confusion matrix*, which is a matrix where each row represents the instances in a predicted class, while each column represents the instances in an actual class. In the case of a binary classification, the 2 × 2 confusion matrix stores the number of elements of class 0 classified as 0, denoted *T*0, and the number of elements of class 0 classified as 1, denoted *F*1. One defines *T*1and *F*0 analogously. In this context, the *Sensitivity TPR *and *Specificity TNR *are defined as follows:

$$TPR=\frac{T0}{T0+F1}$$

$$TNR=\frac{T1}{T1+F0}$$

A ROC plane is a plane where *y *= *TPR *and *x *= *FPR *= 1 - *TNR*, and it is useful to measure a classification in terms of *TPR *and *FPR *rates, once having established to represent with 0 the positive class. Note that, since a classifier assigns data items to classes, the *TPR *represents the percentage of item pairs correctly assigned to different classes, while the *FPR *is the percentage of item pairs incorrectly assigned to different classes. In the ROC plane, it is possible to define the ROC curve, which is a two-dimensional visualization of *TPR *versus *FPR *for increasing threshold values. Indeed, the area under this curve (AUC for short) is defined in the range [0,1], where a value of 0.5 corresponds to the performance of a classifier with a random assignment rule, while the closer is AUC to one, the better is the performance of the classifier. The CROC curve of a distance is a transformation of the ROC curve in which each point corresponds to a proper clustering solution.

We address point (C) by:

(C.1) showing how to map a clustering solution into the ROC plane (see subsection *Clustering solutions, ROC plane and the BMI index*)

(C.2) introducing a distance between a clustering solution and GS (see subsection *A procedure to compare distance functions and clustering algorithms via ROC analysis*);

(C.3) showing how (C.1) and (C.2) can be used to fairly compare the intrinsic ability of distance functions and of a clustering algorithms to identify "structure" in a dataset (see subsection *A procedure to compare distance functions and clustering algorithms via ROC analysis*).

The *BMI *takes values in the range [0,1]. Moreover, the closer the value of the index is to zero, the better the agreement between a partition and the GS. Therefore, in contrast to the most popular external validation indices [27], which need to be maximized, *BMI *needs to be minimized.

## Results

The *BMI *can be regarded as the core of the methodology, and here it has been used in multiple ways: to assess a distance, to assess an algorithm, or purely as an external validation index. In the following, we give the result details for each one of these uses.

Values on Table 1 show the *BMI *computed for the three considered distances on each of the 9 datasets. In this case, the *BMI *refers to the best clustering solution produced, i.e. the closest point to (0,1) on the CROC curve (see the paragraph *The BMI index and the CROC curve *in the *methods *section for details). Therefore, the lower the value of the *BMI *for a distance, the better its intrinsic discrimination ability is. An analysis of the results in Table 1 shows that the Euclidean distance *d*_2 _and Pearson correlation distance *d_r _*have a good intrinsic discrimination ability when computed on most of the datasets (see the bold values in the Table 1), except on CNS-RAT and Yeast, in agreement with [9] and [30]. The same behavior is not confirmed by the mutual information distance *d_MI_*, which does not seem to offer the same flexibility and versatility as the other two distances. Taking into account such a fact, together with the results presented in [10], one receives an indication that *MI *may be superior to the considered distances only in conjunction with clustering algorithms specifically designed for its use. That is, although theoretically superior to other distances in terms of its ability to capture statistical dependency, it does not seem to offer the same flexibility and versatility as the other two distances considered here.

**Table 1:** BMI-values

	*d*_2_	*d_r_*	*d_MI_*
CNS Rat	0.6804	0.6875	0.6692
Gaussian3	0.7170	0	0.7102
Gaussian5	**0.2358**	0.5424	-
Leukemia	0.3498	**0.2559**	0.3000
Lymphoma	0.3509	**0.3385**	0.7028
NCI60	**0.4699**	**0.4699**	0.5643
Novartis	**0.4260**	**0.4240**	**0.4183**
Simulated6	**0.5022**	0.8150	0.7456
Yeast	0.6647	0.6750	0.6677

The *BMI *value for each of the considered distances and for each dataset. For Gaussian5, the *d_MI _*is not computed due to the small number of features. In bold, the values of *BMI *which reveal a good discrimination ability of a distance.

**Figure 1:**
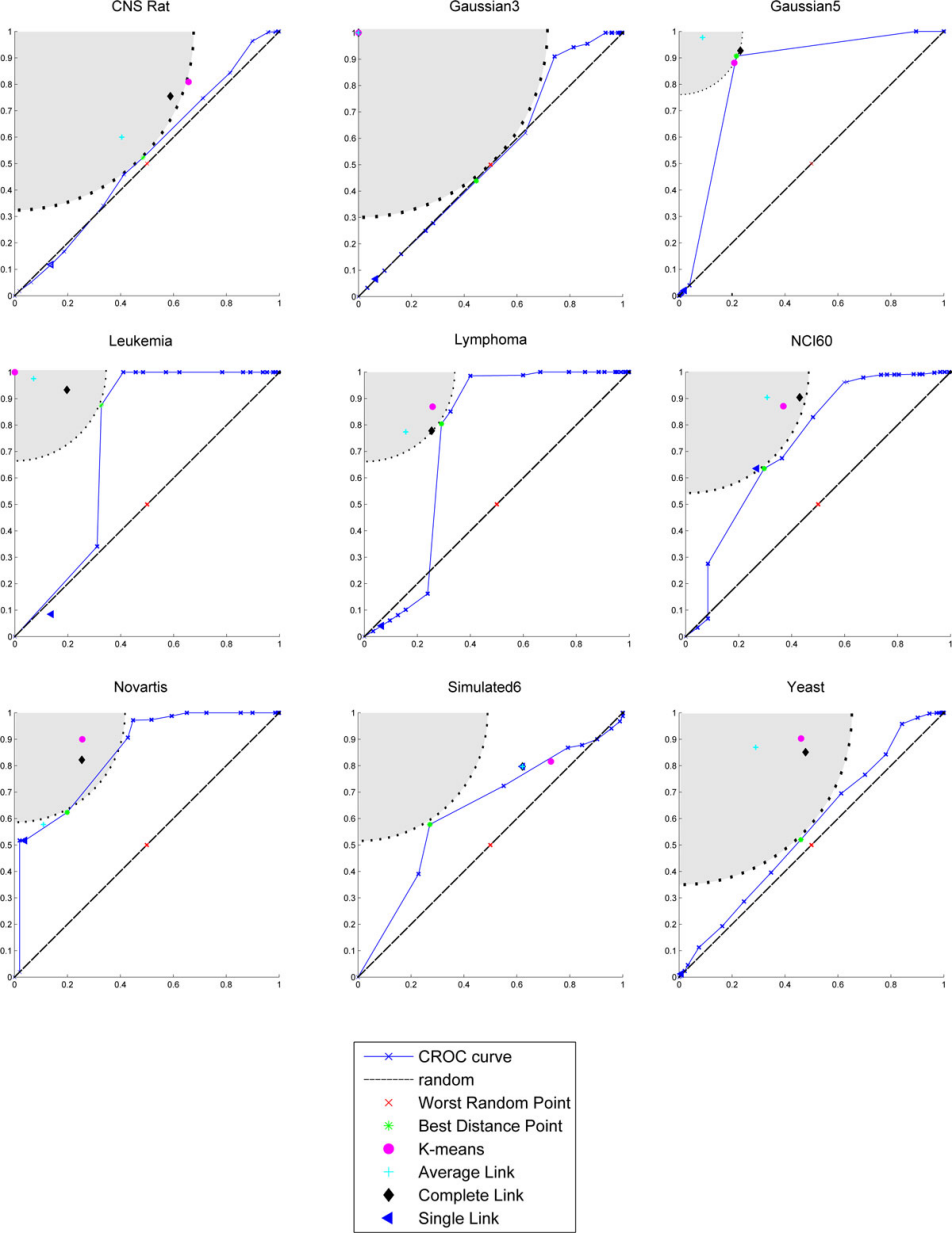
**CROC Euclidean**. The CROC curve and plot of the clustering solutions for each dataset in the case of the Euclidean distance. Each subfigure is referred to a dataset. The markers show *TPR *versus *FPR *of each clustering solution. The area in gray represents the set of points which have a better performance with respect to the best distance point for *BMI*, while the dotted line represents set of points with the same performance. Complete link, K-means and Average link fall into the gray region for most of the dataset.

**Figure 2:**
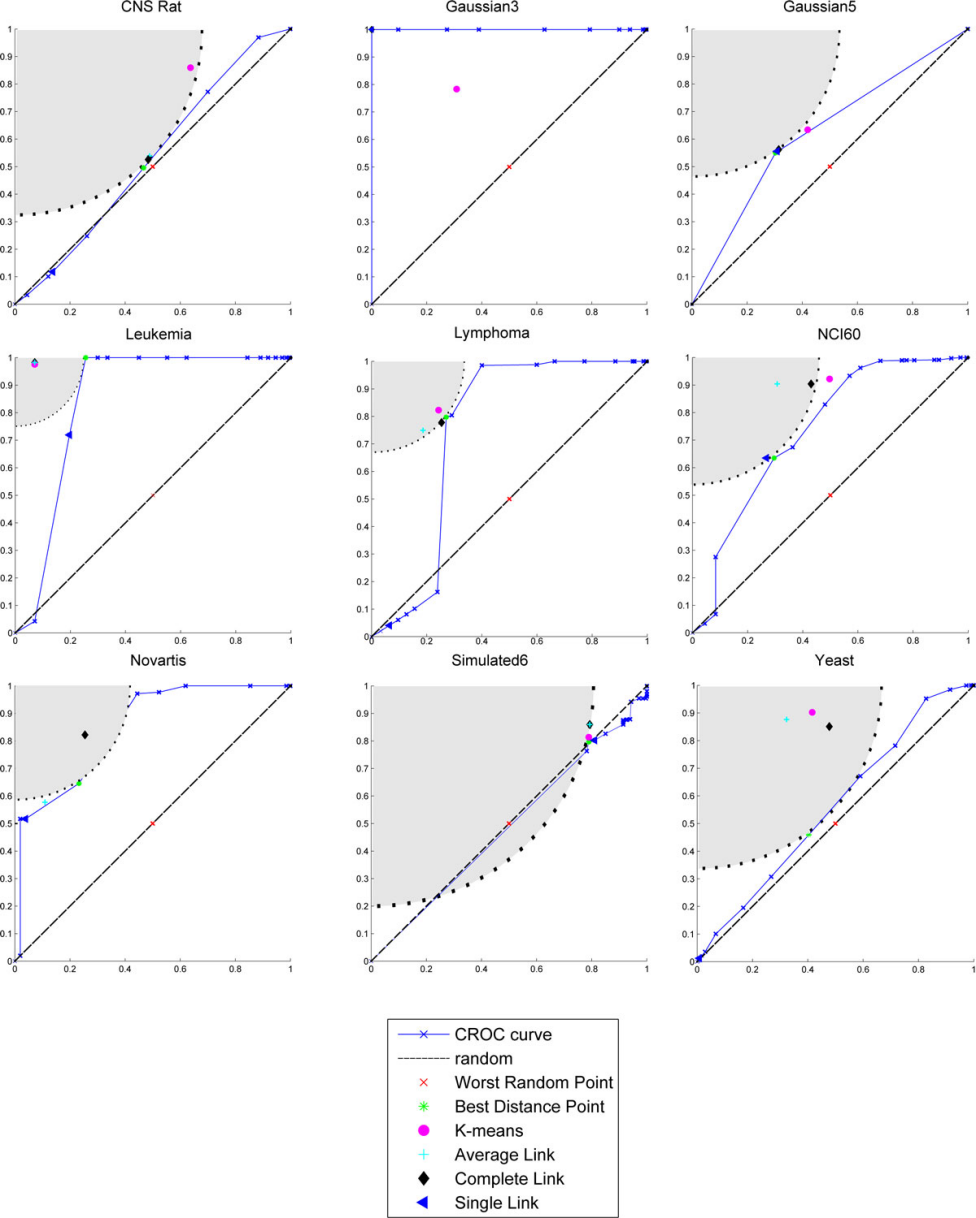
**CROC Pearson**. The CROC curve and plot of the clustering solutions for each dataset in the case of Pearson Correlation. Each subplot is referred to a dataset. The markers show *TPR *versus *FPR *of each clustering solution. The area in gray represents the set of points which have a better performance with respect to the best distance point for *BMI*, while the dotted line represents set of points with the same performance. As in Figure 1, Complete link, K-means and Average link fall into the gray region for most of the dataset.

**Figure 3:**
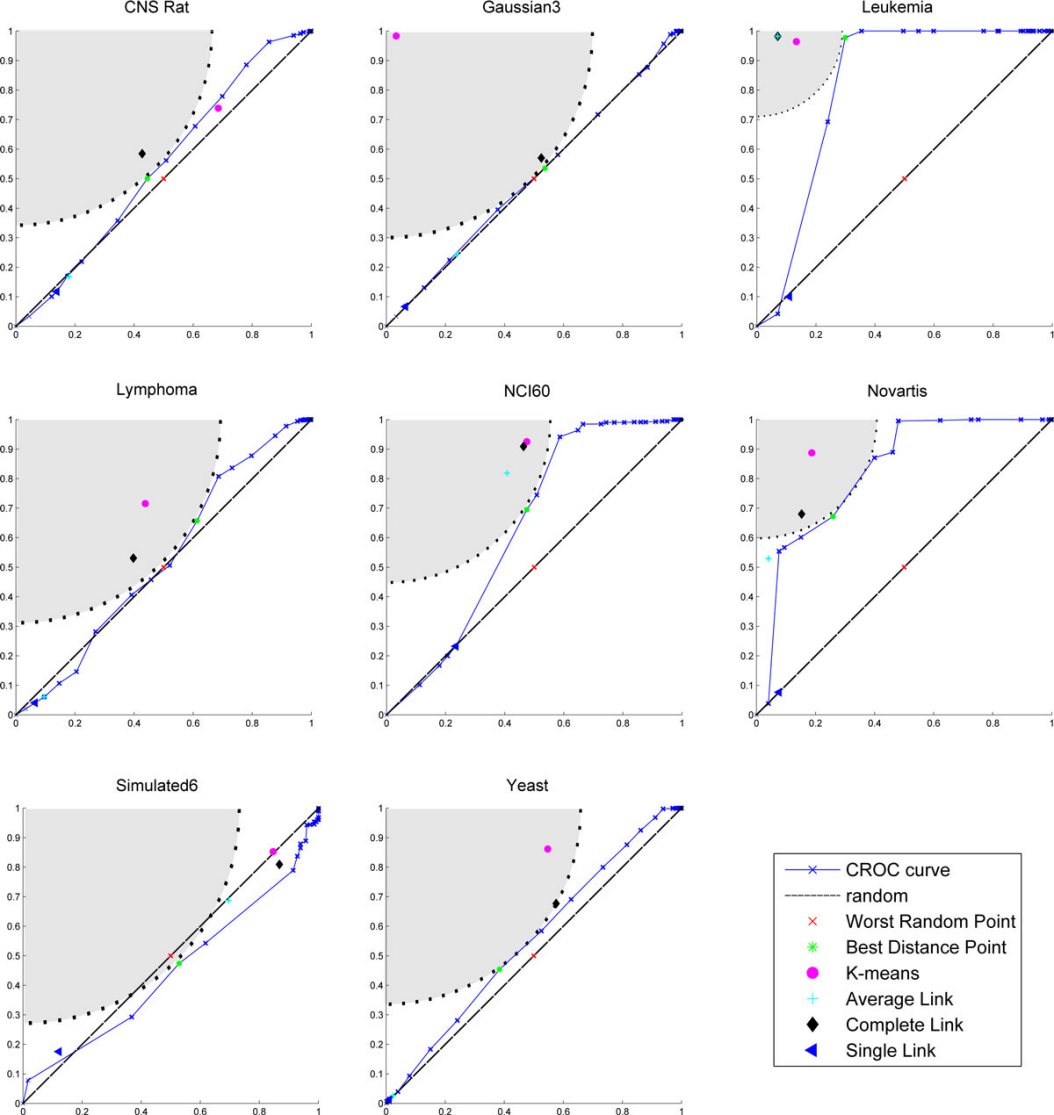
**CROC Mutual Information**. The CROC curve and plot of the clustering solutions for each dataset in the case of Pearson Correlation. Each subplot is referred to a dataset. The markers show *TPR *versus *FPR *of each clustering solution. The area in gray represents the set of points which have a better performance with respect to the best distance point for *BMI*, while the dotted line represents set of points with the same performance. Complete link and K-means fall into the gray region for most of the dataset, but this is not the case of Average Link.

Figures 1-3 show the partitions in the ROC plane for each considered algorithm, and the CROC for each distance. As it is discussed next, the availability of both *BMI *and CROC allows a detailed analysis of the interaction between a distance function and a clustering algorithm and represents one of the main contributions of this research. Indeed, we have that K-means, Average Link, and Complete link clustering algorithms are in most cases able to improve the intrinsic separation ability of a distance function with respect to clustering. This can be observed by looking at the gray area in the corresponding figures since it represents the set of points which have a better performance with respect to the best distance point for *BMI*. Thus, we can assert that an algorithm improves the intrinsic separation ability of a distance when its *BMI *falls inside the grey region. In particular, Complete link is the best performer, falling 19/27 times into the gray region, followed by K-means (16/27) and Average link (12/27). It is worth pointing out that the intrinsic separation ability of *d_MI _*is not improved by Average Link and that Single Link is the worst performer (3/27). Moreover, the mapping of a clustering solution s to a point *P_s _*allows to observe the agglomerative or divisive behaviour of the clustering algorithm that has produced *s *(see subsection *Clustering solutions, ROC plane and the BMI index *for more details about the methodology). Results show a more divisive behavior of Complete Link for all the considered distances, and of K-means in the case of Pearson and MI distances.

**Figure 4:**
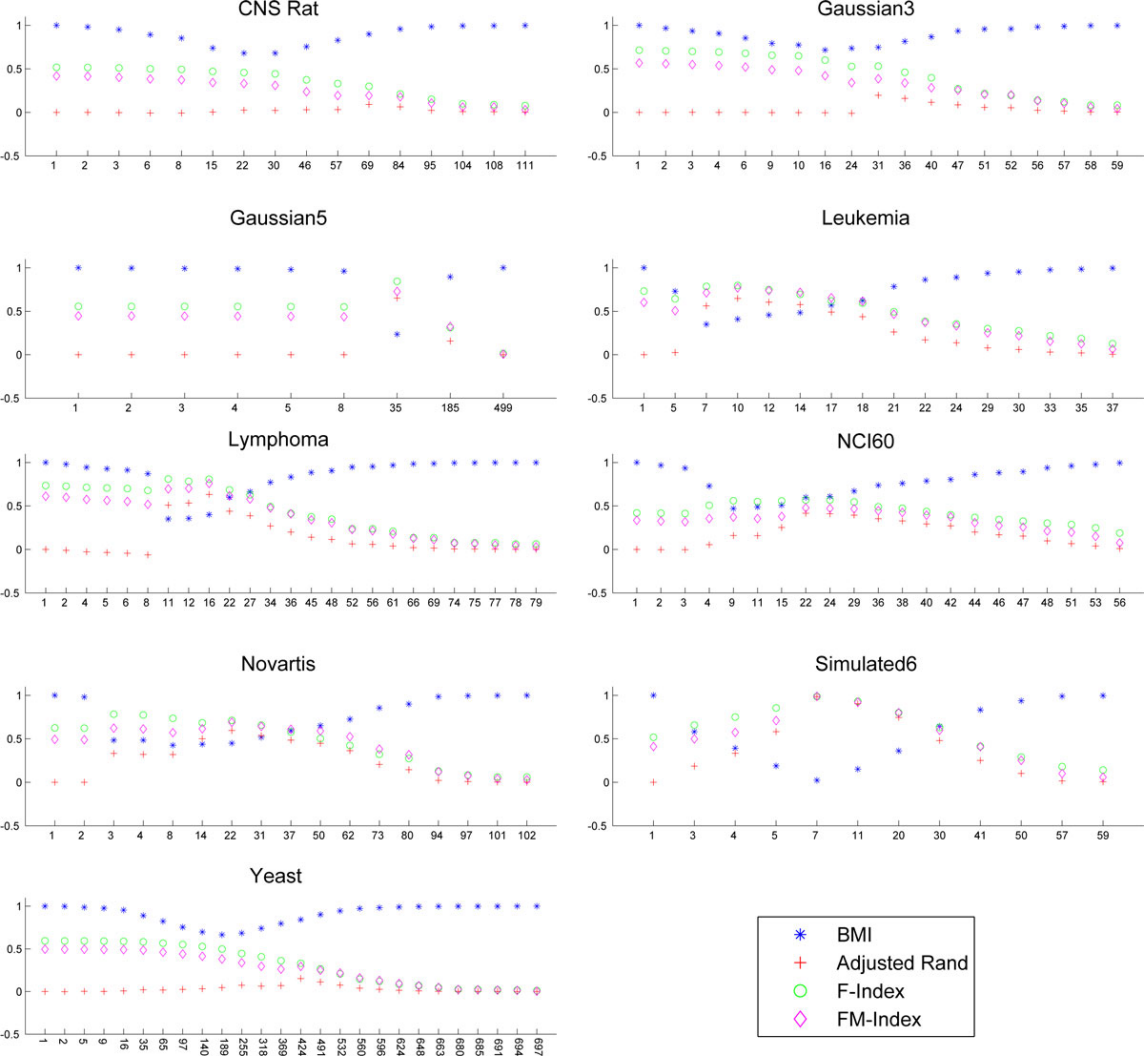
***BMI *and *R_A_*,*F*,*FM *Euclidean**. Plots of the *BMI, R_A_, F, FM *for all the clustering solution corresponding to the points in the CROC of the Euclidean distance. Each subplot refers to a dataset.

**Figure 5:**
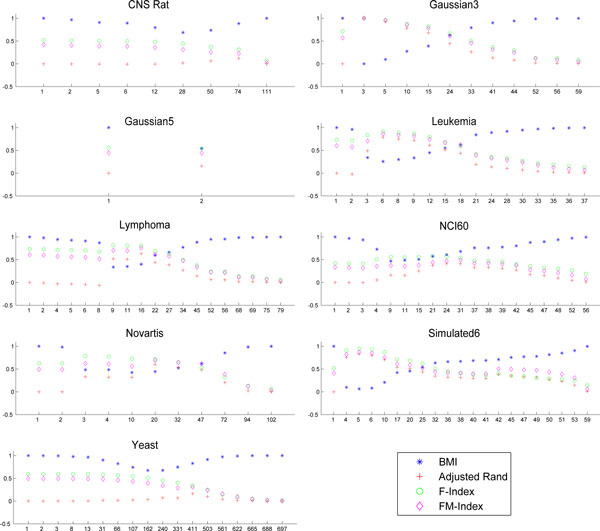
***BMI *and *R_A_,F,FM *Pearson**. Plots of the *BMI, R_A_, F, FM *for all the clustering solution corresponding to the points in the CROC of the Pearson Correlation Distance. Each subplot refers to a dataset.

**Figure 6:**
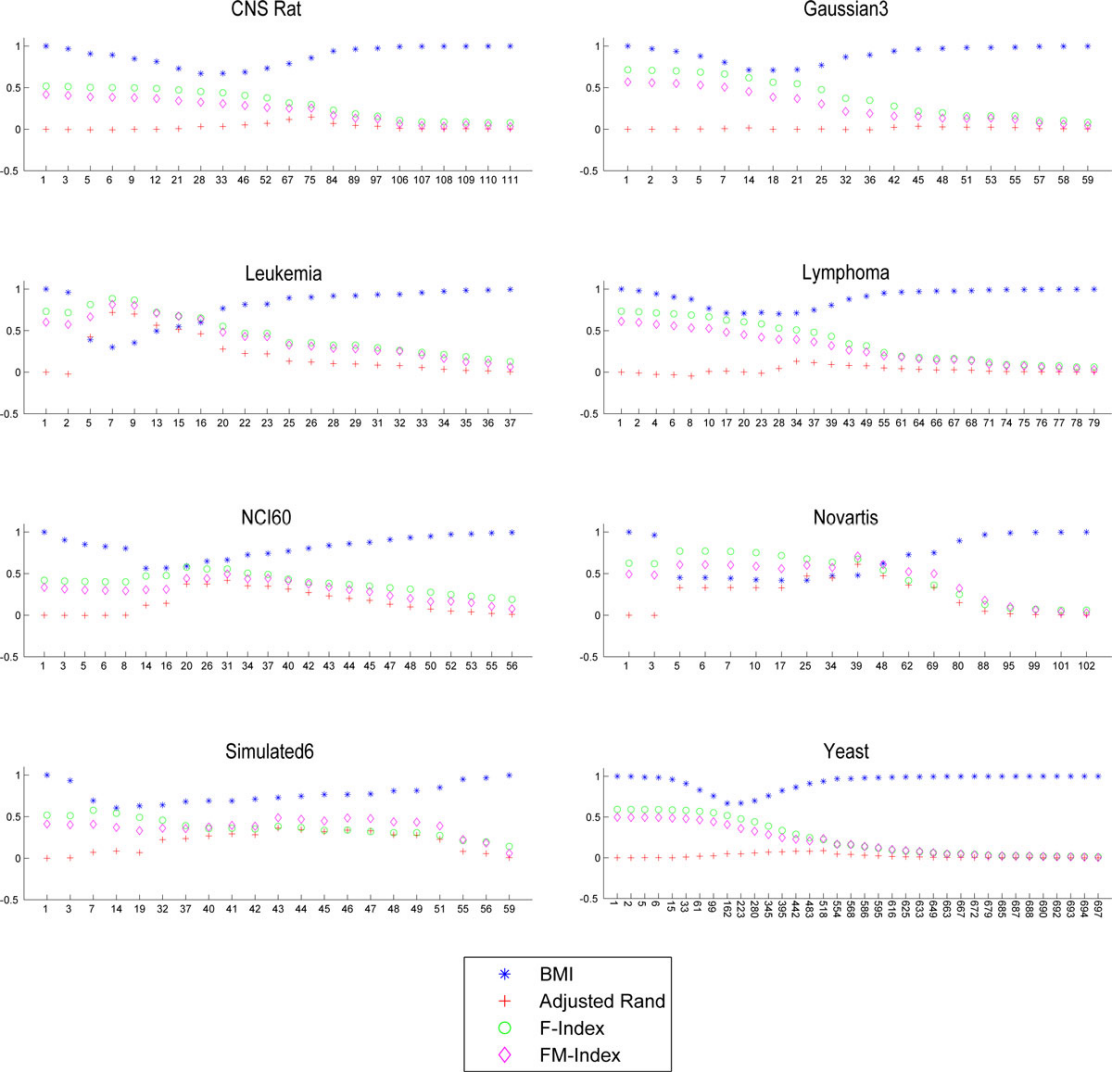
***BMI *and *R_A_,F,FM *MI**. Plots of the *BMI, R_A_, F, FM *for all the clustering solution corresponding to the points in the CROC of the *MI *distance. Each subplot refers to a dataset. For Gaussian5, the *d_MI _*is not computed due to the small number of features and the corresponding subplot has been removed from the figure.

The *BMI *can be regarded also as an external validation index and it can be used to asses the performance of clustering algorithms. Therefore, in order to validate this new index we compare it with the state of the art indices in the literature. Indeed, we have considered the set ${P_{d},}_{X}$ of all the clustering solutions corresponding to the points in the CROC for each considered distance *d*, and each dataset *X*. Then, for each element in ${P_{d},}_{X}$ we have computed the *BMI*, the Adjusted Rand (*R_A_*), the Fowlkes and Mallows (*FM*) and the *F* values. For the convenience of the reader, the latter three indices are defined in the *Methods *section. Figures 4-6 plot the values of *BMI, R_A_, FM, F *for each clustering solutions in ${P_{d},}_{X}$. Each figure refers to a distance, and each sub-figure to a dataset. The x-axis represents the number of clusters of each clustering solution in ${P_{d},}_{X}$. The Figures seem to suggest a strong anti-correlation between the curves of *BMI *and the other indices. In order to quantify this correlation, we have computed the Pearson correlation between the curves of BMI and those latter indices. The results are in Tables 2 - 4 and show that the *BMI *is on most of the datasets highly anti-correlated with the three other indices. In particular, *BMI *shows 13/27 times a strong anti-correlation (bold values in Tables 2) and 10/27 times a moderate anti-correlation with *R_A_*, 13/27 times a strong anti-correlation (bold values in Tables 3) and 12/27 a moderate anti-correlation with *F*, and 11/27 times a strong anti-correlation (bold values in Tables 4) and 13/27 times a moderate anti-correlation with *FM*. This demonstrates that the *BMI *values are congruent with other indices, so its use as an external validity index is fully justified.

**Table 2:** *BMI *vs *R_A_*

	*d*_2_	*d_r_*	*d_MI_*
CNS Rat	-0.1397	-0.2682	-0.3476
Gaussian3	-0.2207	-**0.997**	0.3336
Gaussian5	-**0.9918**	-**1**	-
Leukemia	-**0.9512**	-**0.9830**	-**0.9754**
Lymphoma	-**0.9498**	-**0.9465**	-0.3409
NCI60	-0.6241	-0.6060	-0.6485
Novartis	-**0.8998**	-**0.8787**	-**0.8750**
Simulated6	-**0.9249**	-**0.9800**	-0.4720
Yeast	-0.5121	-0.5106	-0.6246

The Pearson correlation between the *BMI *and *R_A _*values computed on all the clustering solutions mapped in the CROC of each of the considered distances, for each dataset. For Gaussian5, the *d_MI _*is not computed due to the small number of features. The strong correlation values are shown in bold, while the weak correlations in grey.

**Table 3:** *BMI *vs *F*

	*d*_2_	*d_r_*	*d_MI_*
CNS Rat	-0.4590	-0.1684	-0.5910
Gaussian3	-0.5031	-**0.8714 **	-0.5371
Gaussian5	-0.5518	-**1 **	-
Leukemia	-**0.8155 **	-**0.8246 **	-**0.8068**
Lymphoma	-0.6329	-0.5915	-0.5896
NCI60	-**0.86139 **	-**0.8533 **	-**0.8529**
Novartis	-**0.793199 **	-**0.7194 **	-**0.8283**
Simulated6	-**0.9419 **	-**0.9373 **	-0.4966
Yeast	-0.4808	-0.3151	0.5448

The Pearson correlation between the *BMI *and *F *values computed on all the clustering solutions mapped in the CROC of each of the considered distances, for each dataset. For Gaussian5, the *d_MI _*is not computed due to the small number of features. The strong correlation values are shown in bold, while the weak correlations in grey.

**Table 4:** *BMI *vs *FM*

	*d*_2_	*d_r_*	*d_MI_*
CNS Rat	-0.3408	-0.0507	-0.5335
Gaussian3	-0.4027	-**0.91840**	-0.4383
Gaussian5	-0.6297	-**1**	-
Leukemia	-**0.8701**	-**0.8628**	-**0.8453**
Lymphoma	-0.6969	-0.6624	-0.5338
NCI60	-0.6801	-0.6429	-**0.7264**
Novartis	-**0.8194**	-**0.7661**	-**0.8230**
Simulated6	-**0.9280**	-**0.9255**	-0.4584
Yeast	-0.4183	-0.2481	-0.4887

The Pearson correlation between the *BMI *and *FM *values computed on all the clustering solutions mapped in the CROC of each of the considered distances, for each dataset. For Gaussian5, the *d_MI _*is not computed due to the small number of features. The strong correlation values are shown in bold, while the weak correlation in grey.

## Conclusions

In this paper we have presented a procedure to asses the discriminative ability of a distance for data clustering. Such procedure is based on the *BMI*, a new external validation index that has the versatility to be used to asses a distance, to asses an algorithm, or purely as an external validation index. We have applied the overall methodology on 9 datasets, in the case of the Euclidean, Pearson and Mutual Information distances. Some of the computed results agree with other state of the art external validation indices, but with respect to them our procedure is more informative since it can shed light on the bias of the clustering algorithm with respect to a distance. An important thing to stress about the proposed methodology is that, although it was validated in the context of gene expression data obtained by microarray technologies, it is worth pointing out that the proposed methodology is "generic", i.e, it can be applied to other kind of data (e.g. RNA-seq). As a future direction of investigation, we intend to extend the proposed methodology to study a very challenging problem in the field of data analysis, i.e., the quantification of the intrinsic *complexity *of a dataset, defined as: the difficulty for a clustering algorithm to find the correct partition of a dataset.

## Methods

### Definition of distance functions

We now formally define the distances used in this paper.

The *Euclidean *distance belongs to the geometric class of distances, and it is defined as follows:

(2) $$d_{2}\left(\vec{x},\vec{y}\right)=\sqrt{\overset{m}{\underset{i=1}{\sum }}|x_{i}-y_{i}{|}^{2}}$$

where $\vec{x}$ = (*x*_1_,...,*x_m_*), $\vec{y}$ = (*y*_1_,...,*y_m_*).

The *Pearson *distance *d_r _*is a correlation-based distance:

(3) $$d_{r}\left(\vec{x},\vec{y}\right)=1-r=1-\frac{\sum_{i=1}^{m}\left(x_{i}-\bar{x}\right)\left(y_{i}-ȳ\right)}{\sum_{i=1}^{m}{\left(x_{j}-\bar{x}\right)}^{2}\sum_{j=1}^{m}{\left(y_{j}-ȳ\right)}^{2}}$$

where $\vec{x}$ and $\vec{y}$ are the sample means of $\vec{x}$ and $\vec{y}$.

The *Mutual Information distance d_MI _*is an information-based distance so defined

(4) $$d_{MI}\left(\vec{x},\vec{y}\right)=1-\frac{\sum_{i=1}^{m}\sum_{j=1}^{m}p_{ij}log\frac{p_{ij}}{p_{i}p_{j}}}{max\left(-\sum_{i=1}^{m}p_{i}logp_{i},-\sum_{j=1}^{m}p_{j}logp_{j}\right)}$$

where *p_i _*= *P*(*X *= *x_i_*) and *p_j _*= *P*(*Y *= *y_j _*) are the marginal probability mass functions (p.m.f. for short) and *p_ij _*= *P*(*X *= *x_i_*,*Y *= *y_j _*) the joint p.m.f. When dealing with such a distance, the problem is the estimation of the marginal and joint p.m.f., which involves a discretization of the data values, usually done by using binning and histogram based procedures [31].

### Definition of external indices

Recall from [27] that an external index measures how well a clustering solution computed by an algorithm agrees with the gold solution for a given dataset. Formally, let *C *= {*c*_1_,...,*c_r_*} be the partition of the items in dataset **X **into *r *clusters, corresponding to the gold solution for that dataset. Let *P *= {*p*_1_,...,*p_t_*} be an analogous partition, possibly produced by a clustering algorithm.

An external index measures the level of agreement of the two partitions. External indices are usually defined via a *r *× *t *contingency table *T*, where *T_ij _*represents the number of items in both *c_i _*and *p_j_*, 1 ≤ *i *≤ *r *and 1 ≤ *j *≤ *t*.

For our experiment we have used the *R_A_, FM *and *F *indices. We report their formulas next, pointing out that additional details about them can be found in [27].

(5) $$R_{A}=\frac{\sum_{i,j}\left(\begin{array}{c} T_{ij}\\ 2 \end{array}\right)-\frac{\left[\sum_{i}\left(\begin{array}{c} T_{i.}\\ 2 \end{array}\right)\sum_{j}\left(\begin{array}{c} T_{.j}\\ 2 \end{array}\right)\right]}{\left(\begin{array}{c} N\\ 2 \end{array}\right)}}{\frac{1}{2}\left[\sum_{i}\left(\begin{array}{c} T_{i.}\\ 2 \end{array}\right)+\sum_{j}\left(\begin{array}{c} T_{.j}\\ 2 \end{array}\right)\right]-\frac{\left[\sum_{i}\left(\begin{array}{c} T_{i.}\\ 2 \end{array}\right)\sum_{j}\left(\begin{array}{c} T_{.j}\\ 2 \end{array}\right)\right]}{\left(\begin{array}{c} N\\ 2 \end{array}\right)}}$$

(6) $$FM=\frac{\sum_{i,j}T_{i,j}^{2}-N}{\left(\sum_{i.}T_{i.}^{2}-N\right)\cdot \left(\sum_{j}T_{.j}^{2}-N\right)}$$

(7) $$F=\underset{c_{i}\in C}{\sum }\frac{T_{i}}{N}c_{ṡ}x_{p_{k}\in P}\frac{\left[{\left(\sum_{i}\left(\begin{array}{c} T_{i.}\\ 2 \end{array}\right)-\sum_{i,j}\left(\begin{array}{c} T_{ij}\\ 2 \end{array}\right)\right)}^{2}+1\right]\cdot \left(\frac{T_{i,j}}{T_{.j}}\right)\cdot \left(\frac{T_{i,j}}{T_{i.}}\right)}{{\left(\sum_{i}\left(\begin{array}{c} T_{i.}\\ 2 \end{array}\right)-\sum_{i,j}\left(\begin{array}{c} T_{ij}\\ 2 \end{array}\right)\right)}^{2}\cdot \;\left(\frac{T_{ij}}{T_{.j}}\right)+\left(\frac{T_{i,j}}{T_{i.}}\right)}$$

where *T_i. _*= |*c_i_*| and *T_.j _*= |*p_j _*|.

Note that there is a little difference in the range of values of the three indices: while the *FM *and the *F *indices can assume a value in the range [0,1], the *R_A _*may be negative [32]. All three indices need to be maximized, that is, for each of them, the closer the index is to one, the better the agreement between the two partitions.

### The BMI index and the CROC curve

The ROC plane can be used to estimate the similarity between a reference partition and a generic one as follows. The reference partition is mapped to the point (0, 1) in the ROC plane, corresponding to perfect classification. Analogously, the generic partition is mapped to a point in the ROC plane, depending on the number of "misclassified" elements with respect to the reference partition. Then, a distance measure between such a point and (0, 1) gives an indication about the similarity of the partitions. The *BMI *is the Euclidean distance between those two points. Moreover, the mapping of partitions into the ROC plane at the base of the *BMI *can be used to assess the intrinsic discriminative ability of a distance function for clustering by generating the CROC curve and by considering the closest point to (0, 1) on this curve as a clustering solution associated to the distance function. Then, the *BMI *between this point and (0,1) gives the required estimate. In the following subsections, we give details about the *BMI *and the CROC.

#### Clustering solutions, ROC plane and the BMI index

Given a gold solution GS, it is possible to map a clustering solution *s *into the ROC plane as follows:

1. Compute the connectivity matrix *J_s _*for *s*.

2. Starting from *J_s_*, compute the confusion matrix with respect to GS.

3. Use that confusion matrix to compute *TPR *and *FPR *for *s*. Those two variables naturally identify a point into the ROC plane, associated to *s*.

A few remarks are in order. The above approach naturally leads to measure a clustering solution in terms of *TPR *and *FPR *rates. As anticipated, the point into the ROC plane associated with GS is *P_GS _*= (0,1).

Given a clustering solution *s*, let *P_s _*= (*x, y*) be the point in the ROC plane corresponding to it.

The performance of *s *is proportional to the proximity of *P_s _*to *P_GS_*, as we now explain. Let *E_m _*be the *Misclassification error rate *defined as the sum between *FPR *(*x*) and False negative rate (*FNR *= 1 - *y*). That is, *E_m _*is the L1 metric (*d*_1_) computed between *P_s _*and *P_GS_*, i.e., *d*_1_(*P_GS_, P_s_*) = |*x *+ 1 - *y*|. Then, the closer *P_s _*and *P_GS _*are with respect to *d*_1_, the better the clustering solution with respect to *E_m_*.

It is worth pointing out that *P_s _*gives a measure of the agglomerative and divisive tendency of a generic clustering algorithm. Indeed, the greater the *x *value, the more divisive the clustering algorithm is. Analogously, the smaller the *y *value, the more agglomerative the clustering algorithm is. Indeed, we can actually devise an index that measures such a tendency.

Let *E_b _*be the *Balancing error rate *defined as the measures of how much *FPR *and *FNR *are unbalanced. The *BMI *for a generic clustering solution is:

(8) $$BMI=\alpha \times {\left(E_{m}\right)}^{2}+\beta \times {\left(E_{b}\right)}^{2}$$

where the weights *α *and *β *allow to tune the importance between balance and misclassification.

Among all the possible weight combinations, a natural choice for *BMI *is to set *α *and *β *in order to take into an equal account the misclassification error rate *E_m _*and the balancing error rate *E_b_*. This corresponds to the setting *α *= *β *= 0.5, and it is of interest and relevance here to notice that in this case *BMI *corresponds to *d*_2_(*P_GS_*,*P_s_*). That is, the *L*_2 _(Euclidean) metric between the points *P_GS _*and *P_s_*. This means that the closer *P_s _*and *P_GS _*are with respect to *d*_2_, the better the clustering solution, in equal measure (*α *= *β *= 0.5) between misclassification error rate *E_m _*and balancing error rate *E_b_*.

Operationally, once fixed *α *= *β *= 0.5, if one wants to compute the *BMI *of a clustering algorithm producing a clustering solution with *x *= *FPR *and *y *= *TPR*, respectively, one needs only to compute the Euclidean distance between the points *P_s _*and *P_GS _*in the ROC plane. It is obvious that such a technique can also be used to compare the performance of several clustering solutions by considering the Euclidean distances between the associated points into the ROC plane and *P_GS_*.

#### A procedure to compare distance functions and clustering algorithms via ROC analysis

We recall from [9] that starting from a distance matrix *D *and a gold solution GS, it is possible to derive a ROC curve into the ROC plane, as we now briefly outline. Given a threshold value *∅ *∈ [0,1] and the distance matrix *D*, let *I_∅ _*be a matrix in which each entry is defined as follows:

(9) $$I_{\phi }\left(i,j\right)=\left\{\begin{array}{cc} 1 & \text{if }D\left(i,j\right)\leq \phi ,\\ 0 & \text{otherwise}\text{.} \end{array}\right)$$

Therefore, considering all the points corresponding to different threshold values, we obtain the ROC curve for the distance function *d*. If each point in the ROC curve corresponded to a proper partition of the items, i.e., a clustering solution, we could use it to address point (C) (see *Evaluating the performance of distance function via the BMI index and the CROC curve*). Unfortunately, that is not the case, as we now argue. In fact, *I_∅ _*cannot be considered as a connectivity matrix of a dataset partition since it could not satisfy the transitive property (see formula 1). This issue always occurs when three generic items *x_i_, x_j_, x_k _*lie on a straight line at distances *D*(*i, j*) = *D*(*j, k*) = *∅*, involving *I_∅ _*(*i, j*) = 1, *I_∅_*(*j, k*) = 1 but *I_∅_*(*i, k*) = 0. Therefore, in order to properly compare a distance function with a clustering algorithm, via ROC analysis, we need to convert the matrix *I_∅ _*into a matrix *J_∅ _*representing a connectivity matrix of a clustering solution. This can be done in several ways: here we have adopted an approach based on the connected components induced by the matrix *I_∅_*. Intuitively, the process is the following: if *I_∅ _*does not correspond to a partition, i.e., at least two sets *a *and *b *have non-empty intersection, then they are merged into a new set *c *= *a *∪ *b*. This allows to transform the ROC curve associated to a distance function into a new curve in which each point corresponds to a proper clustering solution. We refer to this curve as the CROC of a distance. Recall from [11] that the AUC represents also the probability that a random pair of elements belonging to different classes will be correctly ranked. By analogy, in our case, the AUC under the CROC represents the probability of the following event: two couples (*x, x*′) and (*y, z*) such that *x, x*′ belong to the same cluster while *y, z *to different clusters satisfy the relation *d*(*y, z*) >*d*(*x, x*′), where *d *represents a generic distance. Using the CROC curve, one can find the best clustering solution associated to a distance function with respect to *BMI*, as the closest point *P *to *P_GS _*into the CROC curve (see the green dot marker in Figures 1 - 3 as an example). Note that the value of the area under the CROC increases while the *BMI *of the best clustering solution decreases, thus we expect a low value of the *BMI *for a distance which has a good intrinsic discrimination ability. One can now fairly compare a distance function and a clustering solution produced by an algorithm, in terms of their classification ability:

1. Compute the ROC curve for a distance function *d*.

2. Calculate the CROC curve starting from the ROC curve computed in the previous point.

3. Find the best point into the CROC curve, i.e., the point with the lowest value of *BMI*, and mark it.

4. Map one or more clustering solutions in the ROC plane (as described in subsection *Clustering solutions, ROC plane and the BMI index*) and mark the corresponding points.

5. Rank the performance of each marked points in the ROC plane, as described in subsection *Clustering solutions, ROC plane and the BMI index*.

## Competing interests

The authors declare that they have no competing interests.

## Authors' contributions

All authors participated in the design of the methods and of the related experimental methodology. LP and FU implemented all of the algorithms and performed the experiments. RG and GL coordinated the research and wrote the report. All authors have read and approved the manuscript.

## Declarations

The publication costs for this article were funded by the corresponding author's institution

This article has been published as part of *BMC Bioinformatics *Volume 14 Supplement 1, 2013: Computational Intelligence in Bioinformatics and Biostatistics: new trends from the CIBB conference series. The full contents of the supplement are available online at http://www.biomedcentral.com/bmcbioinformatics/supplements/14/S1.
